# Supplementary material for: Next generation reservoir computing
Source: Nat Commun. 2021 Sep 21;12:5564. doi: 10.1038/s41467-021-25801-2 (PMC8455577; doi:10.1038/s41467-021-25801-2)
Supplement: Supplementary file 1 — Supplementary Information [file 41467_2021_25801_MOESM1_ESM.pdf]

Supplementary Information

Next Generation Reservoir Computing

Daniel J. Gauthier,[1,2,*] Erik Bollt,[3,4] Aaron Griffith,[1] and Wendson A.S. Barbosa[1]

[1]The Ohio State University, Department of Physics, 191 West Woodruff Ave., Columbus, OH 43210, USA.

[2]ResCon Technologies, LLC, PO Box 21229, Columbus, OH 43221, USA

[3]Clarkson University, Department of Electrical and Computer Engineering, Potsdam, NY 13669

[4]Clarkson Center for Complex Systems Science (C^3S^2), Potsdam, NY 13699, USA

*Correspondence to: gauthier.51@osu.edu

September 8, 2021

Supplementary Note 1. Forecasting Verification

For the Lorenz63 system, we use two methods for verifying that the forecasted attractor is an accurate representation of the true attractor. The first relies on comparing the unstable steady states of the predicted and true attractors and the second is a qualitative comparison to the return map associated with the strange attractor. For the double-scroll system, we only compare the true and predicted unstable steady states.

Lorenz63 Unstable Steady States

The unstable steady states (USSs) of the true Lorenz63 system are determined by setting the derivatives in Eq. (7) to zero and solving for the values of the three variables. There are three solutions given by

$$\mathbf{X}_{uss} = [0,0,0]^T,\ [\pm\sqrt{(8/3)(28-1)}, \pm\sqrt{(8/3)(28-1)}, (28-1)]^T. \quad (1)$$

We calculate the L_2 (Euclidean) distance from the predicted USSs to their corresponding true value. To allow easy comparison of the accuracy of these USSs, we calculate these distances in a uniformly scaled space where Lorenz63 has unit variance. For the model predictions used to generate the attractor shown in Fig. 2, the L_2 distance from the zero USS, calculated in a uniformly scaled space where the Lorenz63 system has unit variance, is $1.2\pm1.4\times10^{-3}$, and the distance between the predicted and true positive (negative) USS is $12.1\pm3.1\times10^{-4}$ ($7.6\pm1.5\times10^{-4}$).

Double-Scroll Unstable Steady States

We find the USS by setting the derivatives in Eq. (8) to zero and solving for the state variables. This reduces to solving the transcendental equation

$$0 = V_1/R_2\,(R_1 - R_4 - R_2) + 2R_1 I_r \sinh(\beta(1 - R_4/R_1)V_1). \quad (2)$$

For the parameters we use, this yields three solutions, and therefore three USSs given by

$$\mathbf{X}_{uss} = [0, 0, 0]^T, [V_1, \pm V_1 R_4/R_1, \pm V_1/R_1]^T. \quad (3)$$

Because the NG-RC for this task has only odd polynomial powers and no constant term, it is symmetric about the origin and predicts the zero USS exactly. The L_2 distance from the true non-zero positive USS, calculated in a uniformly scaled space where the double-scroll system has unit variance, is $2.1 \pm 0.2 \times 10^{-3}$.

Return map

The z variable of the Lorenz63 system has a functional relation between successive local maxima. This is demonstrated visually by finding the successive local maxima M_i of z, and then plotting M_i with respect to M_{i+1}. This return map neatly summarizes the long-term behavior of the z variable and comparing two such maps provide a quick way to qualitatively compare two systems and has been used previously to verify that a trained RC can replicate the Lorenz63 climate.

To evaluate whether an NG-RC can replicate this long-term behavior, we perform the same procedure on a free-running forecast produced by an NG-RC previously trained on the Lorenz63 system. For both the NG-RC and Lorenz63, we look for maxima in window of 1,000 time units.

The values of the maxima in the discrete-time solutions for both the NG-RC and Lorenz63 depend on the time step dt used for integration, as the true maximum may be achieved in between the discrete time steps. To better reproduce the true Lorenz63 return map and to reduce the effect dt has on the NG-RC return map, we interpolate the z solutions by using a degree-4 spline. The local maxima are then found on this interpolated spline.

Supplementary Figure 1 shows both return maps. Qualitatively, there is good agreement between the two return maps. The NG-RC return map almost completely obscures the true Lorenz63 return map at this scale. Upon close inspection, we see that the NG-RC return map does not line up precisely with the true Lorenz63 return map. This can be improved by extending the training time of the NG-RC, but difference between the two return maps is already small even when the NG-RC is trained for only 10 time units (400 total data points).

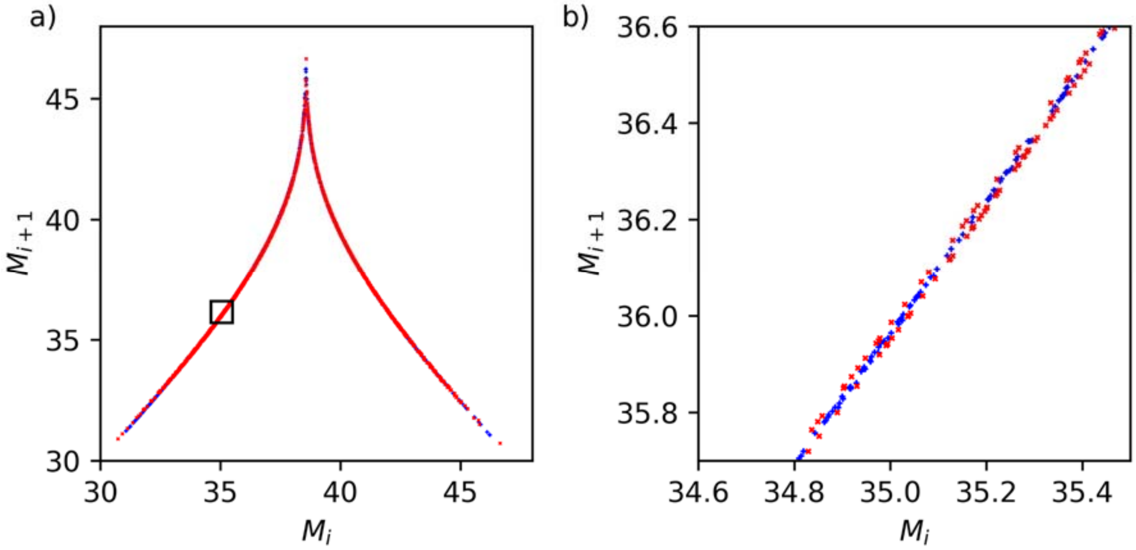

Supplementary Figure 1. a) The z return map of Lorenz63 (blue +) overlaid with the z return map of the NG-RC forecast (red x). The NG-RC reproduces the long-term dynamics of the z variable accurately enough at this scale that it is difficult to see the true return map underneath. b) Detail of the region marked in a).

Supplementary Note 2. Elements of $\mathbf{W}_{out}$ for the two tasks

Forecasting task

Supplementary Figure 2 shows the x, y, and z components of $\mathbf{W}_{out}$ for the forecasting task presented in Fig. 2 of the main text and Supplementary Figure 3 shows a zoom-in of the components. These components vary smoothly with the regularization parameter α over the range we consider in this work. In comparison to the vector field of Eq. 7, there are many substantial components that do not appear directly in the vector field that result from stepping the vector field forward in time, which defines the flow.

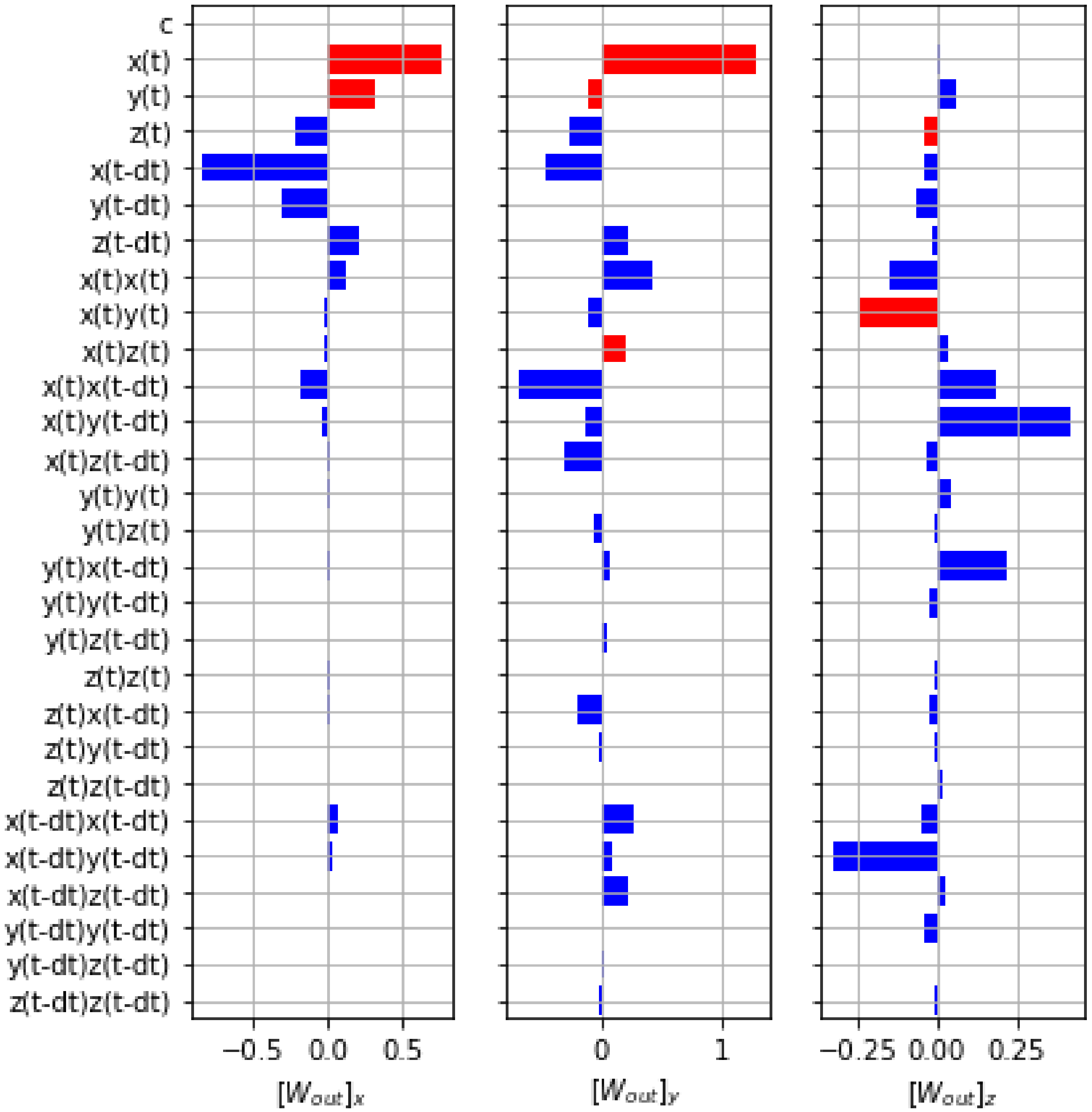

Supplementary Figure 2. Elements of the training output weight matrix for the forecasting task. The red (blue) bars indicate terms that are found (not found) directly in the vector field.

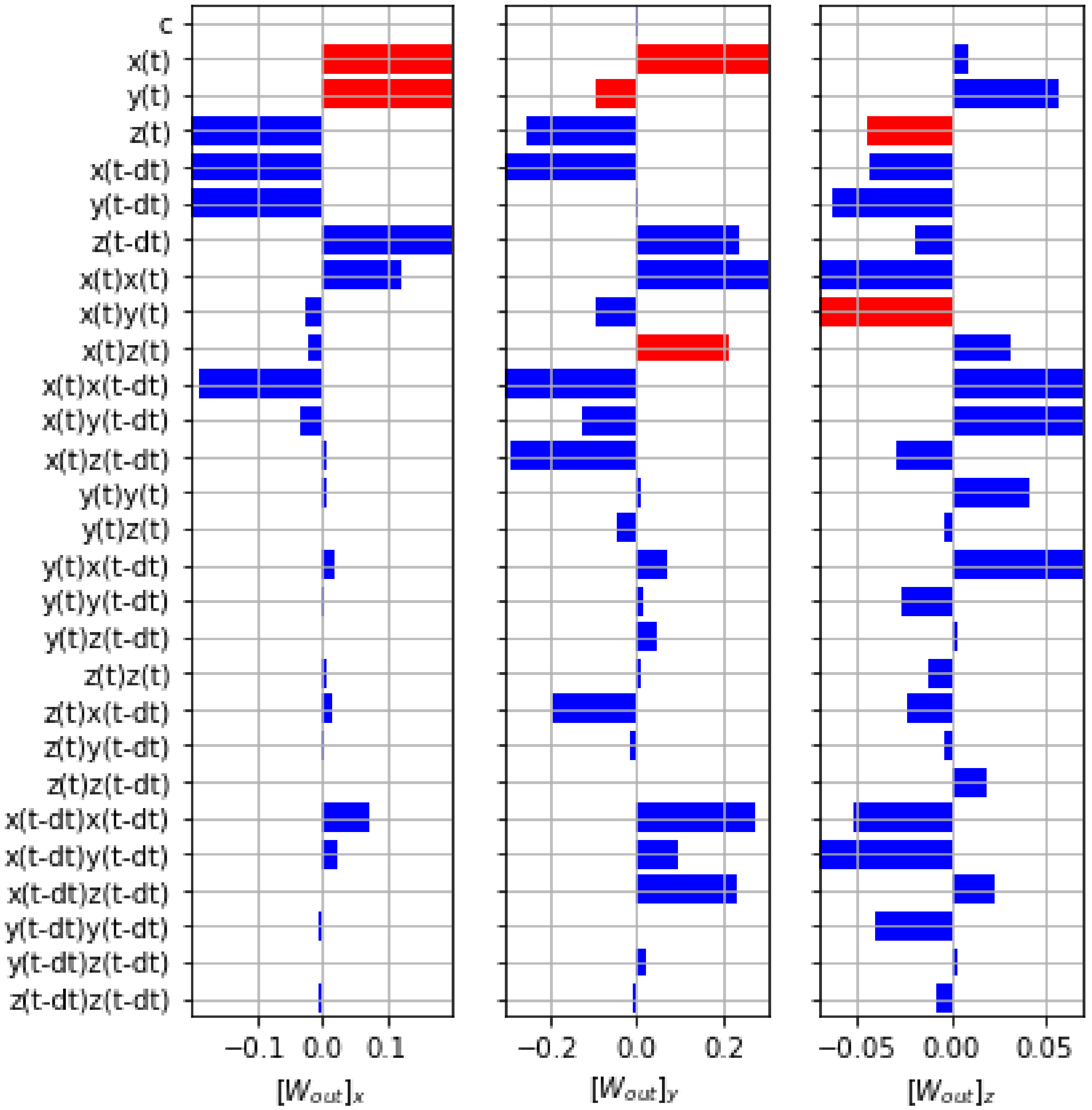

Supplementary Figure 3. Zoom in of the elements of the training output weight matrix for the forecasting task. The red (blue) bars indicate terms that are found (not found) directly in the vector field.

Inference Task

Supplementary Figure 4 shows the components of the output weight matrix for the inference task presented in Fig. 4, where the z is inferred given x and y. The largest component of $\mathbf{W}_{out}$ is the constant term c. It can be explained by the fact that z has a considerable offset as shown in the time series of Fig. 2 of the main text, although all the other $\mathbf{W}_{out}$ components are non-zero and contribute for the final output to some extent.

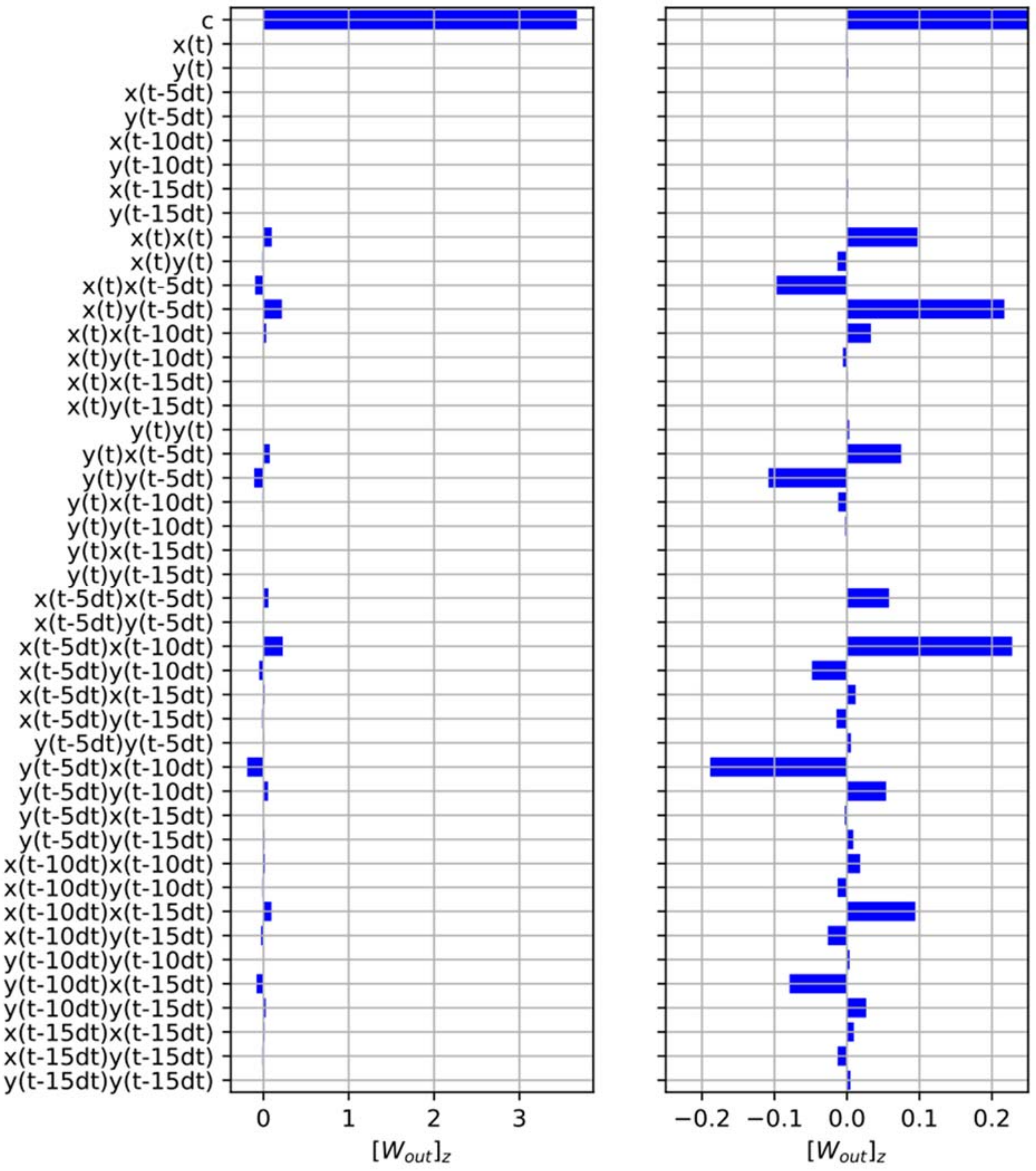

Supplementary Figure 4. (left) Elements and (right) a zoom in of the elements of the training output weight matrix for the inference task.

Supplementary Note 3. Comparing the computational complexity of the NG-RC with a typical traditional RC

Here, we provide an indication of computational speed up for the NG-RC compared to a traditional RC for the Lorenz63[3,12,14] (quartic nonlinear output layer for the NG-RC) and double-scroll[12] (cubic nonlinear output layer for the NG-RC) forecasting tasks by estimating the number of multiplications and special function evaluations for each. Our assumptions and notation are as follows, where we only track parameters that contribute the most to the computational complexity.

NG-RC and traditional RC parameters:
Warm-up steps: M_{warmup}
Training steps: M_{train}

NG-RC-specific parameters:
Dimension of the linear part of the feature vector: N_{linear}
Multiplications need to form feature vector: $N_{nonlinear}$
Total components in the feature vector: N_{total}

Traditional RC-specific parameters:
Number of reservoir nodes: N
Sparsity of the internal reservoir connections: σ_r
Number of special function evaluations (typically tanh): $N_{special}$

Supplementary Tables 1 and 2 give our estimates of the computational complexity of these examples. For the traditional RC, we assume sparse connectivity and that there is no additional overhead for using sparse matrix multiplication routines. Special function evaluations can be computationally expensive or not depending on built-in mathematical co-processors. We give the number of these evaluations but do not use it in comparing the computational complexity.

The dominant contribution to the computational complexity for the NG-RC is performing the ridge regression, which is $\mathcal{O}((M_{train}(N_{total})^2)$ over the training time. The dominant contribution for the traditional RC is multiplying the reservoir state with the adjacency matrix, which is $\mathcal{O}(\sigma_r (M_{warmup}+M_{train}) (N_{total})^2)$ over the warmup and training time, and performing the ridge regression, which is $\mathcal{O}(M_{train}(N_{total})^2)$ over the training time assuming all nodes contribute to the prediction. Also, we have $N_{special} = N$, but we do not consider this computational cost.

We estimate the speed up by summing the dominant contributions for the traditional RC and divide by the sum for the NG-RC. The traditional RC used in Ref. 12 is meant to be efficient (fast simulation time) at the expense of some accuracy, the one used in Ref. 14 is meant to have high accuracy at the expense of longer simulation time, while the one used in Ref. 3 is intermediary. Clearly, our analysis indicates a substantial speed up even with our conservative analysis, while the NG-RC simultaneously obtains high accuracy.

Supplementary Table 1:
Estimated speed up of the NG-RC for the Lorenz63 forecasting task

	M_{warmup}	M_{train}	$N_{nonlinear}$	N_{total}	N	σ_r	speed up
NG-RC	2	400	21	28	-	-	-
Ref. 12	1,000	1,000	-	100	100	0.01-0.05	33-163
Ref. 3	? (set to 0)	5,000	-	300	300	0.02	1.5×10^3
Ref. 14	10^5	6×10^4		4,000	2,000	0.02	3.2×10^6

Supplementary Table 2:
Estimated speed up of the NG-RC for the double-scroll forecasting task

	M_{warmup}	M_{train}	$N_{nonlinear}$	N_{total}	N	σ_r	speed up
NG-RC	2	400	56	62	-	-	-
Ref. 12	1,000	1,000	-	-	100	0.01-0.05	8-41

Supplementary Note 4. NG-RC performance with training data set size

We do not yet have an analytic expression for predicting the required training data set size for an RC. We hypothesize that a lower bound on the training data set size is about the number of unknown fit parameters, equal to the number of features times the dimension of the forecasted system (3 in our examples). For the NG-RC and the Lorenz63 task, this is 84 (see Supplementary Table 1), whereas it is 12,000 for Ref. 14. This is approximately the minimum number of data points needed so that the model passes exactly through the training data points. To generalize the model to unseen data, as required for forecasting, requires some additional data overhead.

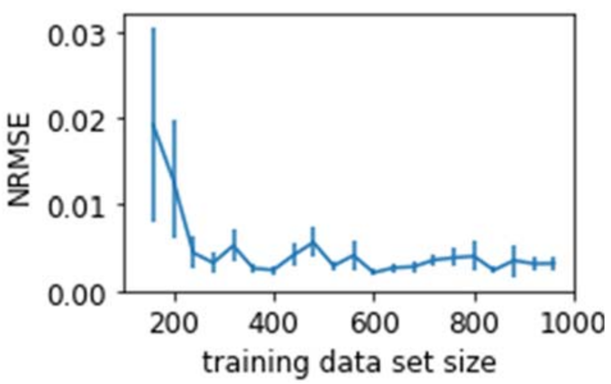

Supplementary Figure 5. NG-RC performance with training data set size for the Lorenz63 forecasting task corresponding to Fig. 2.

Supplementary Figure 5 shows the training data set size dependence of the testing NRMSE, averaged over 20 different temporal segments, for $\alpha=2.5\times10^{-6}$ for all cases. For low training data, there are large fluctuations in the error and there is greater sensitivity to changes in α. Around 250 training data points, the error saturates, indicating that no more data is needed for good, generalized performance. The NRMSE is only one measure of performance; we find that the return map, discussed above in Supplementary Note 1, is more sensitive to the training data set size and we find small, improved performance beyond the 400-point set used in the main text.

We stress that the training data set size required for good performance also depends on the sampling step size dt. If dt is too small, only a small region of the attractor is visited for a small number of data points. If dt is too large, higher-order nonlinear features might be needed. Our

choice of dt, which gives about 40 points per Lyapunov time, balances these two constraints and leading to a small, required data set.

Supplementary Note 5. NG-RC performance for the Lorenz63 system driven by noise

Here, we explore the ability of the NG-RC when a dynamical system is driven by large noise. Here, we augment the differential equations for the Lorenz63 system by adding Gaussian random noise to the right-hand-side of the differential equations for each variable with a root-mean-square value of 1. This noise level is about 12% of the typical root-mean-square values of each variable, which are equal to 7.9, 9.0, and 8.6 for x, y, and z, respectively.

To obtain good generalization, we find that we need to increase the ridge parameter to $\alpha = 1.4\times10^{-2}$. The Lorenz63 dynamics and NG-RC fit are shown in Supplementary Figures 6a)-d).

After the model is trained, we use the NG-RC to forecast the Lorenz63 dynamics initialized by the last point in the training data set. We then compare the predicted behavior to the noise-free Lorenz63 dynamics as shown in Supplementary Figures 6e)-h) and find that the RMSE = 1.34×10^{-2}. This low error indicates that the NG-RC learns the underlying deterministic system even when it is perturbed by substantial noise.

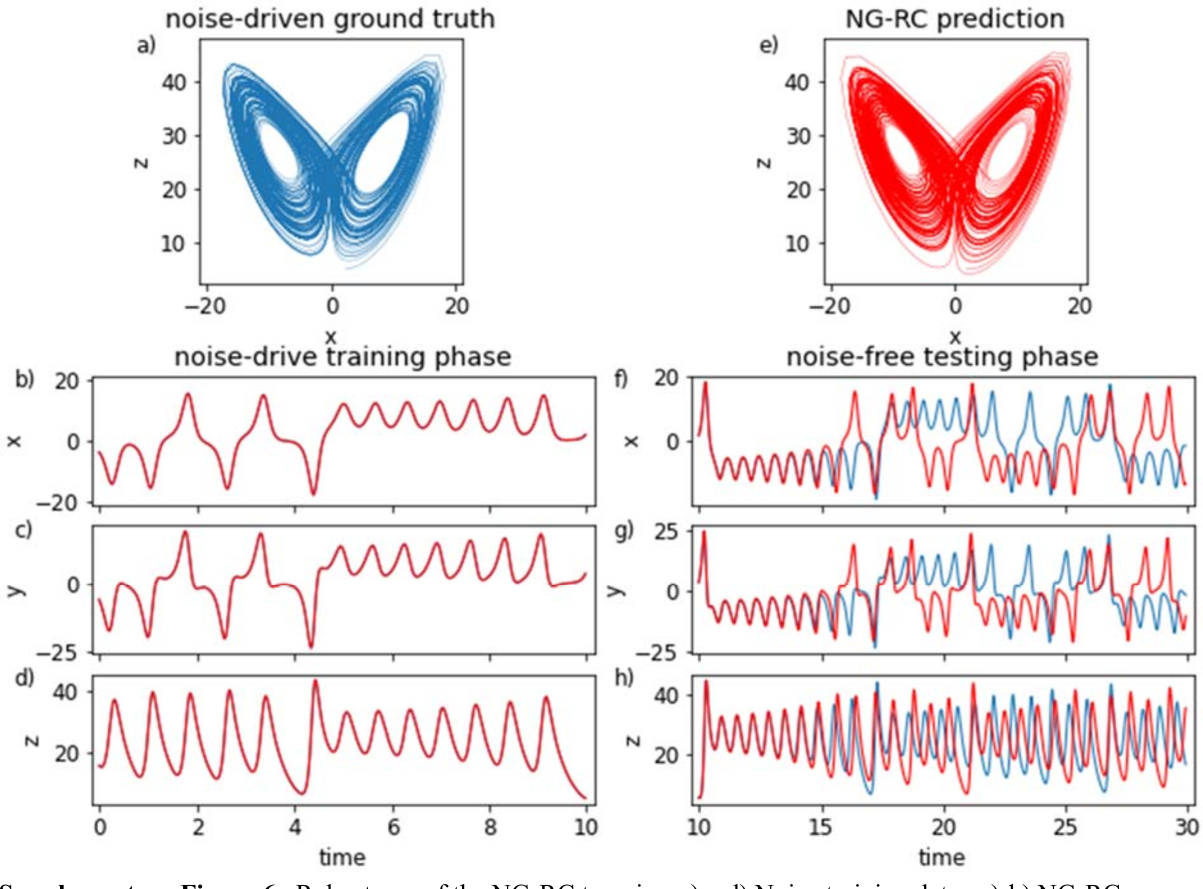

Supplementary Figure 6. Robustness of the NG-RC to noise. a) – d) Noisy training data. e)-h) NG-RC prediction compared to the noise-free ground-truth behavior.
